# Future of industry 5.0 in society: human-centric solutions, challenges and prospective research areas

**DOI:** 10.1186/s13677-022-00314-5

**Published:** 2022-09-08

**Authors:** Amr Adel

**Affiliations:** Yoobee College of Creative Technologies, School of Technology, Auckland, New Zealand

**Keywords:** Industry 5.0, Human machine collaboration, Supply chain, Disaster recovery, Smart healthcare, Cognitive systems, Green manufacturing

## Abstract

Industry 4.0 has been provided for the last 10 years to benefit the industry and the shortcomings; finally, the time for industry 5.0 has arrived. Smart factories are increasing the business productivity; therefore, industry 4.0 has limitations. In this paper, there is a discussion of the industry 5.0 opportunities as well as limitations and the future research prospects. Industry 5.0 is changing paradigm and brings the resolution since it will decrease emphasis on the technology and assume that the potential for progress is based on collaboration among the humans and machines. The industrial revolution is improving customer satisfaction by utilizing personalized products. In modern business with the paid technological developments, industry 5.0 is required for gaining competitive advantages as well as economic growth for the factory. The paper is aimed to analyze the potential applications of industry 5.0. At first, there is a discussion of the definitions of industry 5.0 and advanced technologies required in this industry revolution. There is also discussion of the applications enabled in industry 5.0 like healthcare, supply chain, production in manufacturing, cloud manufacturing, etc. The technologies discussed in this paper are big data analytics, Internet of Things, collaborative robots, Blockchain, digital twins and future 6G systems. The study also included difficulties and issues examined in this paper head to comprehend the issues caused by organizations among the robots and people in the assembly line.

## Introduction

A major shift comes through the first industrial revolution (Industry 1.0) in the eighteenth century, where items were being produced by means and processes invented and allowed to be produced by machines. It started in England in 1760 and reached the United States by the end of the eighteenth century. Industry 1.0 marked a shift from the handicraft economy to dominate by machinery and impacted the industries such as mining, textile, agriculture, glass, and others [1]. The next shift to the manufacturing industry from 1871 and 1914 is termed Industry 2.0, which allowed for faster transfer of persons and innovative ideas. This revolution is a period of economic growth, increasing business productivity causing a surge in unemployment as machines replace factory workers.

**Fig. 1:**
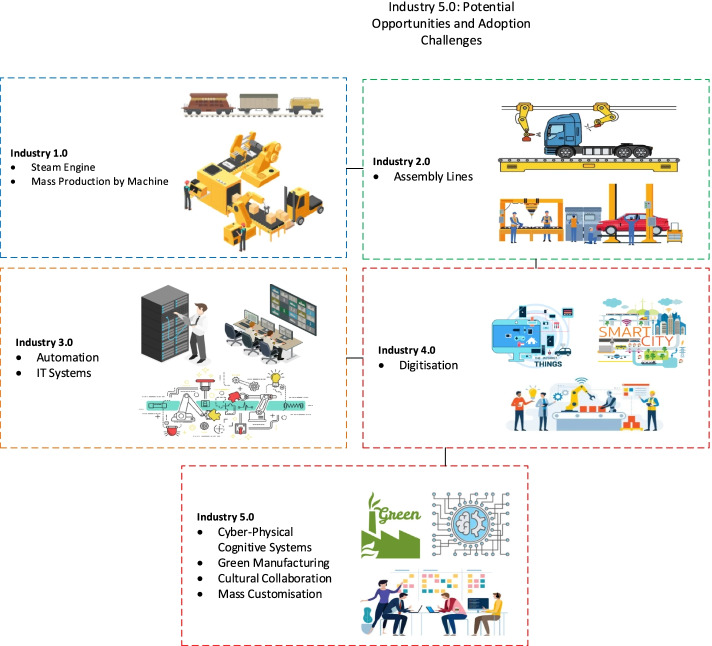
Industrial Evolution from Industry 1.0–5.0

Industry 3.0 is termed the digital revolution, started in the 70s in the twentieth century through the automation of memory-programmable controls plus computers. The central point of this particular phase is mass production and the use of digital logic, integrated circuit chips; derived technologies included computers, digital cellular phones, and the internet [2, 3]. The innovations of the technology are transforming traditional products as well as business procedures. The digital revolution is converting technology into digital format. Industry 4.0 is a union among the physical assets and advanced technologies such as artificial intelligence, IoT, robots, 3D printing, cloud computing, etc. The organizations that adopted 4.0 are flexible and prepared for data-driven decisions [4]. Industry 5.0 is the upcoming technology of the previous generation designed for efficient and intelligent machines. Figure 1 shows the industry revolution from industry 1.0 to industry 5.0. Table 1 remarks the most relevant surveys that discuss some aspects of industry X.0.

**Table 1:**
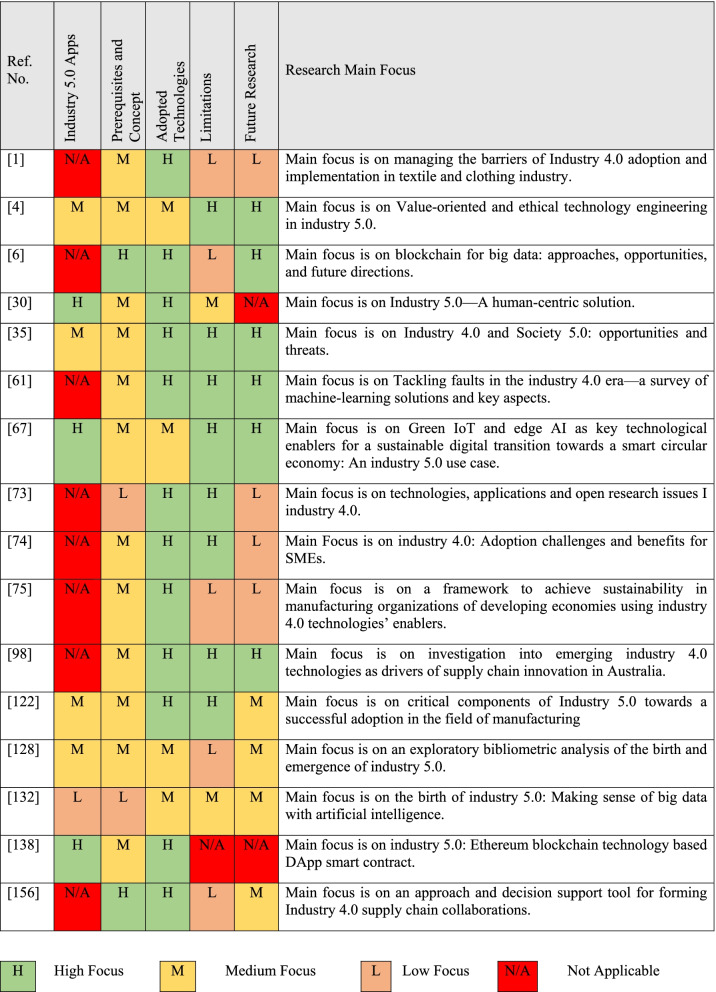
Summary table of most relevant surveys

Note: references within the table [1, 5–19]

### The rationale following the progression of industry 5.0

The revolution of industry 5.0 means that humans and machines are working together, improving the efficiency of industrial production. Human workers and universal robots are boosting the productivity of the manufacturing industry [5]. Each of the executive teams of the manufacturing company is required to define the production line, then follow the key performance indicators and ensure that the processes are working effortlessly. The future direction of industry 5.0 is the manufacturing of robots and industrial robots. The advancement of artificial intelligence and cognitive computing technologies is taking the manufacturing world to a high speed and increasing business efficiency [20]. Apart from the benefits in the manufacturing business, industry 5.0 also benefits in sustainability as it aims to develop a sustainable system that runs on renewable energy.

In order to adopt industry 5.0 for the companies, the personnel are required proper interaction among the machines as well as operators. It is knowledge in the fields like robotics as well as artificial intelligence [21, 22]. The role of the business organization is based on making decisions around the advanced factors. Training employees is required with virtual education to decrease the cost for the businesses, as it is not required production to stop for providing training to the employees. It provides safe training that can prevent the workers from being uncovered to needless issues during the training sessions. Communication and employee motivation are boosted by resulting in interactive knowledge environments [1, 6, 23, 24]. The employment positions are related to communication with the robotics systems as well as artificial intelligence.

Collaborative robots are being designed for intuitive interaction with humans. Expansion of the digital twins is required technology in industry 5.0. Visual models of the products, processes, and generation will allow better understanding and testing. The Nexus Integra platform is the software required to drive the transformation of the industrial business in industry 5.0 [7]. It is an integrated system for the large-scale management of industrial assets, allowing companies to leap towards digital transformation. Previous generations adapted lifestyles to what machines can do [25, 26]. Still, industry 5.0 differs from all previous resolutions, as humans are at the present face with the center in production procedures.

### Contributions of the paper

The significance of the survey has set to defining the definitions and features of the fifth generation from the literature sources that can help understand the term industry 5.0 from the perspectives of various authors. There is also a discussion of various features of Industry 5.0 as compared to the industrial evolutions. Moreover, there is a discussion of the applications to develop and enable in industry 5.0 like the healthcare, supply chain, production in manufacturing, cloud manufacturing and others. The key technologies of industry 5.0 are also discussed in this paper, including big data analytics, Internet of Things, collaborative robots, Blockchain, digital twins and future 6G systems. Finally, this paper also discusses the challenges to understand the issues related to robots and humans in manufacturing factories. There is a highlighting of the future direction of the research work towards the realization of Industry 5.0.

### Structure of the paper

In section 2, there is reviewing of the definitions of Industry 5.0 from the literature sources with added features of 5.0 in comparison with the past industrial revolutions. The applications are also discussed in this paper in section 3. Later, the enabling technologies for industry 5.0 are discussed in section 4, such as big data, IoT, cloud computing, 6G networks, Blockchain. Section 5 highlights. Finally, in section 6, the conclusion is provided with summarizing the entire paperwork.

## Explanations and modernization

### Explanation of definitions

i. The term Industry 5.0 refers to people working with robots and smart machines. It is about robots helping humans work faster by leveraging advanced technologies such as big data analytics [27, 28].
ii. Industry 5.0 is termed as the revolution in which man and machine are findings ways to work for improvement means and efficiency of the manufacturing production [29].
iii. Ocicka and Turek [30] suggested that Industry 5.0 is compelled with various industries technologists, philosophies, and others to focus on the human factors and technologies in the manufacturing systems.
iv. Industry 5.0 is considered the edge of the smart factory, where it communicates with robots and humans [13, 31]. It uses social networks for communication purposes among humans and electronics components.
v. Industry 5.0 added human-centric, sustainable, and resilient concepts to the industrial revolution. It will revolutionize the manufacturing systems worldwide by preventing repetitive tasks from human workers [32]. The intelligent robots will penetrate manufacturing supply chains as well as the workflow of the production to unparalleled levels.

### Added features of industry 5.0

Industry 5.0 is taking over the past improvements, and it is an effective process due to its highest level of perfection, and the machine work reduces the time and effort of the human workers [33]. Apart from the challenges, a few features encourage business organizations to implement industry 5.0. For instance, in the medical sector, the professionals are working on in the direction of creating a synthetic pancreas. This project has not yet finalized. The sufferers who diagnosed with Type-1 diabetes have been provided with monitoring device that checks blood and levels of sugar in their blood. This device is interconnected with another device that has the capability to deliver the insulin into the body. This is one of creative technologies that has been developed and personalized for patients in terms of providing a reliable and handy control system for the patient. Industry 5.0 is taking this personalization to the next level as it enables the medical doctors to provide the patients with an application that they can install on their smart phones, so they can be traced by their lifestyle and daily routing and a customized plan can be made for them. This would be a life-changing for the Type-1 diabetes sufferers, as the technologies utilized are based on artificial intelligence (AI) systems. These AI systems have the capability to understand and learn the different reactions of the body and act accordingly.

Increase the maintenance plan: Predictive maintenance is required for the smart sensors, IoT, customized software as it requires proper monitoring and maintaining of the failures in the smart devices. The machines will probably break down, and a maintenance plan will stop it [34, 35].

Sustainability: Industry 5.0 is promising to use the resources adjusted to the current requirements of the manufacturing industry. Collaboration among humans and machines leads to supple business models. Waste along with overproduction is to be reduced to eliminate it. Along with new efforts, local production makes economics sustainable [8, 36]. With industry 5.0, corporate technologies are changing the trend. It leads to the emergence of sustainable policies, like minimal generation of the waste and management that can make the companies as effective. Industry 5.0 is created to be applicable through purposefully concentrating on creative research as well as setting knowledge at the frontline of the evolution. It is considered as being marked by a determination that is more than just manufacturing goods for profit. The fundamental principles of Industry 5.0 are: sustainability, human-centricity, and resilience.

The efficiency of humans and productivity: Advanced technologies bring people back to the production center. Collaborative robots perform repetitive and dangerous jobs while people focus on creativity and efficient business solutions [37]. The skills are led to an increase in business productivity, where people feel motivated to do the work and receive the results. A human-centered methodology highlights human demands over the manufacture procedure. Producers have to recognize what technology can do for the people and focus on how technology can adjust to the requirements of the worker instead of the other way. It is essential that technology tackle autonomy and privacy issues.

Environmental control: Smart and connected sensors and customized software provide a real-time predictive overview of the climate, temperature, consumption of energy, and others [38]. It is helpful for business firms to prevent losses and improve production. For maintaining the sustainability of the manufacturing process, it must improve iterative procedures that repurpose, recycle and recover assets. Environmental influence has to be decreased. Sustainable manufacturers can utilize the developed technologies, for example, artificial intelligence to boost personalization, which minimize waste and optimize source-productivity.

Forecast line production efficiency: Smart and connected machines, machine learning, industrial automation is forecasting the efficiency of the production based on the existing activity. It increases business efficiency, where the processes are to be adjusted based on the parameters to avoid losses [39]. Manufacturers need to improve a higher level of resilience in production to enhance and defend their industrial production against disturbances and disasters such as covid-19.

Creativity: Technological innovations are not allowing for a degree of personalization that can meet with demands of the customers. Personnel is part of industry 5.0, which can leverage the potential of the technology [40]. It finds ways to provide new ideas that can lead to product development with personalization in mind.

### Modernizations and innovations

Industry 5.0 is evolving in different domains such as healthcare, manufacturing, textile, education, food, and others. The products are discussed by Bundesgartenschau [41], a woollen pavilion with the robot hand developed by a joint venture of the businesses. KR 500 FORTEC robot is being used as the product. It can perform various types of carpenter tasks like moving the components, applying adhesives, and enabling the robots to collaborate.

By adopting industry 5.0, most industries are moving towards the smart social factory. The project selected to better understand the concepts of industry 5.0 is an intelligent management project of Repsol. The business is employed in the Blockchain, robotic processes automation technology to enhance the security and productivity of the business. The automated guided vehicle is the first Cobot of Repsol that carries out the logistics works such as deposition of waste, delivery of the raw materials from warehouse and lab visualization. Repol is conducted on the project Block lab, where the business is transmitting sensitive data through the property of Blockchain [42–47]. The project is designed to streamline the samples of safety issues, and it is properly managing 10,000 samples every year.

## Industry 5.0 creative applications

### Smart hospital

Industry 5.0 is aimed to create a smart hospital with real-time capability. The technology can provide remote monitoring systems within healthcare [48]. It plays a key in making life better for the doctors. In the COVID-19 pandemic, doctors can use this smart healthcare technology to focus on infected patients and provide efficient data regarding better treatment [1, 49]. Even it also helps the students as well as medical students for needed medical training through the outbreak of COVID-19. Machine learning (ML) is applied to medical imaging, natural language processing, plus genetic data [7]. It is focused on the diagnosis of the diseases, detection, plus prediction of the diseases.

Abdelmageed and Zayed [50] mentioned that industry 5.0 allows manufacturing of the personalized smart implant properly as per the change in requirements of the customers. The medical professional is moving towards artificial intelligence technology to measure various problems like glucose levels. It helps implement mass personalization by producing implants per the patent match, which are the initial needs for orthopedics [51]. Even there is a change in the traditional method manufacturing of the implants of patients, and it is also capable of upgrading various medical devices plus tools. The technologies are used in revolution as it is helpful to perform the surgery in a precise manner [10, 52]. It is helpful for medical students to provide better education, learning, and research and expansion procedures [53]. In orthopedics, industry 5.0 requires high-quality implants with an extended life that is personalized. It helps solve various challenges like over-production, wring selection of the tool and lack of transparency [35, 54, 55].

### Manufacturing industry

**Fig. 2:**
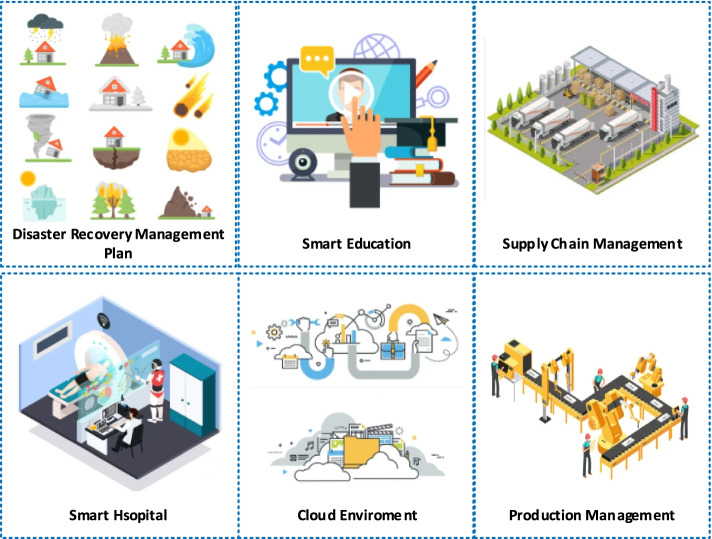
Applications of Industry 5.0

Industry 5.0 is considered a new production model where it is focused on interaction among humans as well as machines. Industry 5.0 is involved in leveraging collaboration among increasing accurate machinery plus the innovative potential of human beings. In order to make manufacturing sustainable, it develops processes that repurpose and recycle the resources [56, 57]. There is also required to reduce the environmental impacts in the manufacturing industry. Additive manufacturing is required to increase personalization to optimize resource efficiency and waste. Industry 5.0 is revolutionizing the manufacturing systems across the globe by taking away repetitive tasks from human workers. Brown and Wobst [58] illustrated that intelligent robots and systems are penetrating supply chains and manufacturing shop floors to an unparalleled stage. Smart manufacturing allows designers to protect design files of manufacturing items by storing them in the cloud with robust access control and usage of the manufacturing resources across various places [59]. Figure 2 pictorially illustrates a number of potential applications within industry 5.0.

Ghobakhloo et al. [60] defined that the designers are permitted to place the manufacturing plants close to raw material and the areas with low manufacturing costs. Control of machines in the plant and operations of the manufacturing lifecycle is to be handled by cloud manufacturing [61]. The service-oriented model helps manufacturing integrate production abilities with the services to provide the clients with proper solutions. Through the business innovations, adding service factors to the production process aims to improve production efficiency, value-added, and the market share for the manufacturing business [9]. The cloud-based platform controls the manufacturing services, and it is used in a cost-optimized way. Cloud manufacturing is networked as well as a distributed system for the production resources.

### Supply chain management

Nguyen et al. [62] determined that supply chain 5.0 highlights the importance of collaboration among smarter machines like COBOTS and humans [63]. Industry 5.0 is aimed to cater to hyper-personalization moreover hyper-customization requirements of the customers, which require combine of human originality plus the competence of the machines. Robots are required for the supply chain management in standardized procedures in high production volumes, added this to each product, and it is a challenge where the robots are required proper guidance [64]. Babamiri, Bahari and Salimi [40] mentioned that the human touch is not required to customize and personalized products. Still, it also ensures seamless end-to-end processes of the supply chain, such as selecting the raw materials to comprehend its personalization and customization needs for the individual consumers. Industry 5.0 seeks to take automated and intelligent digital ecosystems and pair them with the human touch [65, 66]. There is leveraging of human elements in such a process that it helps customize the end-user experiences and optimized workflows.

Human intelligence is worked with the empowered way with cognitive computing along with intelligent automation abilities to enable hyper-personalization. The technologies like machine learning, robotic automation, and others are helping the employees increase business proficiency and deliver high value to the customers faster [12, 67]. From delivering the raw materials, transactions, transportation, the ERP system manages the supply chain for the business organization [68]. The next generation of supply chain solutions is making and deploying the technology to empower the digital supply chain. It means bringing customization to the supply chain, improving the customers’ satisfaction and the management of the business efficiency and market margins. There is the reduction of the risks related to supply chain and wastages based on the existing information of the business. Ietto et al. [32]concluded that there is the improvement of the supply chain integration for the strategic partnerships and enabled the supply chains to spend time on the experimentation plus less on the fighting forces on matters for the project executions.

## Industry 5.0 technologies

The enabling technologies related to industry 5.0 include cloud computing, Blockchain, analytics of big data, IoT and 6G networks.

### Cloud computing

**Fig. 3:**
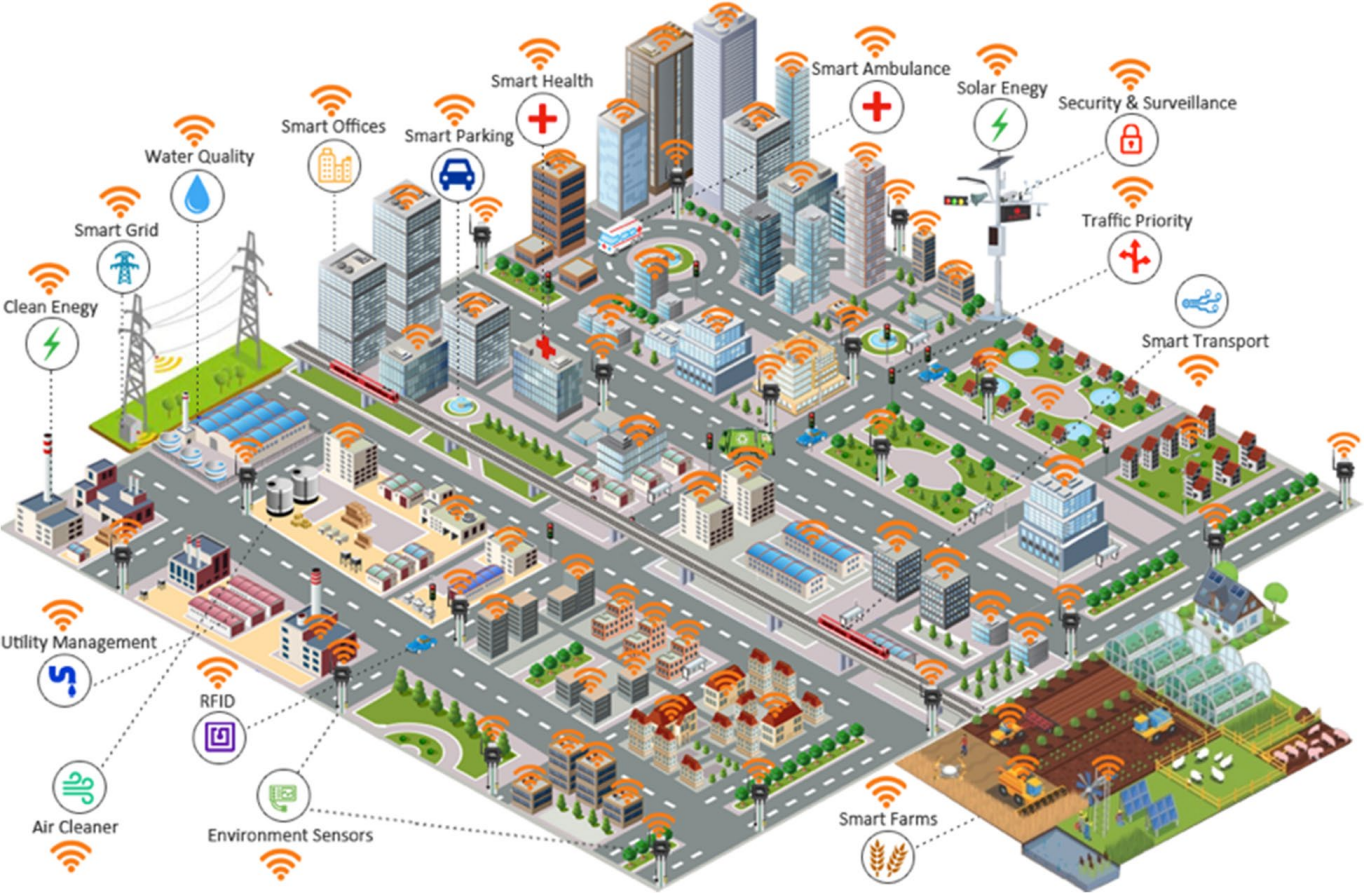
Cloud Ecosystems in Critical Sectors

Cloud computing is the delivery of computing services those are included databases, software, intelligence analytics, networks, and others [69]. This technology is offering efficient innovation and economics of the scale. This technology uses the internet to store and manage data on the remote servers, and then data is accessed via the internet. It delivers on-demand computing services from applications to storage plus processing power [58, 70]. The industrial cloud is the virtual environment that provides a supportive environment for industry applications [71–73]. The cloud providers are manufacturing applications like IoT monitoring tools adopted for mobile and web usage. The cloud also supports the usage of API that can automate data normalization from diverse data production sources [74]. Edge computing devices handle data analytics equipped with limited computing resources to manage the business analyses. Global cooperative type of typical cloud ecosystems in critical sectors is pictorially demonstrated in Fig. 3.

According to Haleem, Javaid and Khan [75], cloud computing provides a scalable infrastructure to support data edge devices. The cloud infrastructure backs edge IoT platforms. The platforms are being used to manage the edge devices like autonomous robots and diverse robots deployed on the shop floor. In order to manage critical data, the industry is access to the data from the local servers daily. Industry 5.0 can reduce the volume of data sent to the centralized server [76]. Cloud computing allows preventive data to detect machine failures and mitigates them by continuing with more workforce.

### Collaborative robots

Industry 5.0 aims to put the human touch back in development and production. It grants the human operators with benefits of the robots like technical precision and heavy lifting abilities [77]. There is a high ability of humans to perform critical tasks, allowing the introduction of a high degree of control and the capability to individualize the production phases [78]. One of the significant implications of collaborative robotics as well as Industry 5.0 is required for human inputs that can extend the existing iterations. Collaborative robots, as well as industry 5.0, are representing new age in robotics plus production. Industry 5.0 plus Cobots is the heart that can combine people’s creativity and craftsmanship with the efficiency and constancy of the robots [62, 79, 80]. From people-centric, the customized products and specialist skills are made more available. Industry 4.0 is focused on ensuring consistency of the quality and data collection.

In contrast, industry 5.0 focuses on highly skilled people plus robots to create individualized products from smart devices to cars for consumers [81]. With industry 5.0, the robots are started to work together. The collaborative robots are accomplished with the tasks of heavy lifting plus ensured consistency, while skilled humans are provided cognitive skills of the craftsperson [82, 83]. It is to be expected that robots are changing relations among humans plus machines in the context of production.

Industry 5.0 refers to people working with robots and smart machines. Robots are helping humans work better by leveraging advanced technologies such as the Internet of things. It added a human touch to industry 4.0 for automation plus business efficiency [11, 75, 84–86]. It is described as a network of physical objects and things embedded with sensors, software, and other technologies. It is a way to connect and exchange data with the devices plus systems over the internet. Ghobakhloo et al. [60] mentioned that IoT is a new paradigm that is to be changed the traditional way to live in the high-tech lifestyle. The research is done to enhance the advanced technologies through IoT. Even IoT is considered a way to provide efficient solutions to data and information security problems. There is the development of secured interaction among social networks plus privacy issues, as it is a hot topic in IoT developers [87]. The smart city is considered an important area for IoT as it incorporates smart homes. It contains IoT-enabled home appliances, heating systems, security systems, and others communicating with each other to provide better comfort and reduce energy [30].

Sinclair et al. [77] concluded that authentication plus access control are the issues in IoT; those are required to have promising solutions to have strong security. A solution is required to verify communication parties to decrease the loss of sensitive data. It is provided with an authentication scheme and verifies various security threats such as man-in-the-middle attacks the key security controls. The proposed authentication and access control approaches help to provide authenticity plus confidentiality end-to-end latency in IoT based on the communication network. It is a dynamic approach for the data-centric applications concerning the cloud platforms.

### Big data analytics

Industry 5.0 is an innovative technology that enables utilizing 3D symmetry in the innovation ecosystem designs. Matheus et al. [70] mentioned that big data analytics is a complex procedure to examine big data to uncover data like hidden patterns, trends of the markets and others. It uses an advanced analytic method with diverse data sets, including structured and semi-structured data. It has massive data sets to store and process through traditional tools [15, 88, 89]. It is used as real-time data to enhance the competitive advantages of the business industry, focusing on providing possible recommendations on predictive discovery. Big data analytics is used to recognize discrepancies while the organization is leveraging a list of the root causes of the issues. Most businesses use big data analytics to make strategic decisions [90–92]. The business uses various factors like population, location accessibility and others to get details of the customer preferences. There is the improvement of the customer experiences by monitoring the customer experiences and addressing problems solutions to build strong customer relationships [93, 94]. Even big data is a challenge for industry 5.0 when detailed information is not gathered on the manufacturing cycle.

### Blockchain

It is decentralized and distributed technology, where the digital ledger contains records named as blocks to record the transactions data. It is a shared ledger that can facilitate recording the transactions and tracking the assets in the business network [95]. The business is running on the information. Therefore, Blockchain technology delivers the data by providing shared and completed information stored in the immutable ledger that the network members access [96–99]. Blockchain technology helps the customers by tracking the orders, payments, production, etc. The network participants have distributed ledger records of transactions, which are recorded to avoid duplication of efforts and records in the database system [100]. In order to speed up the transactions, a smart contract is stored on Blockchain and is to be executed on an automatic basis. It is defined as conditions for the corporate, including terms for paid travel insurance [100–102]. The transactions are to be blocked in irreversible chains, and it strengthens verification of previous blocks plays the entire Blockchain transaction is done. Data accuracy is required for the business to validate the transactions, which are recorded [103–105]. With the distributed ledger, network members share, so time wastage is eliminated.

### 6G and beyond

It is a sixth-generation standard for developing wireless interactions technologies that can support cellular data networks. 6G organizations are relied upon to display significantly greater heterogeneity than their ancestors [106]. They will probably help applications past current portable use situations, like virtual and increased reality (VR/AR), omnipresent moment correspondences, and inescapable knowledge of the Internet of Things (IoT). Normally, versatile organization administrators will embrace adaptable decentralized plans of action for 6G, with nearby authorizing, range sharing, framework sharing, and wise mechanized administration supported by versatile edge processing, artificial brainpower, short-parcel correspondence and Blockchain advances [107]. For industry 5.0, 6G networks are expected to meet the intelligent information society standards to deliver ultra-high reliability [108]. Artificial intelligence methods are used to get mobility predictions solutions to ensure network connectivity. The challenge of industry 5.0 included a high data rate for various applications [109]. As large smart devices are connected, energy management is an issue for industry 5.0. There is the optimization of energy management through the usage of energy consumption plus methods of energy harvesting.

## Challenges of industry 5.0

With around industry 5.0, it is easier to overlook the potential challenges. The challenges are being identified and solved for industry 5.0 developments to succeed for the business.

1. People are required to develop competency skills, as working with the advanced robots, the human workers are required to get knowledge about collaboration with the smart machine and robot manufacturer [110]. Apart from the soft skills required, gaining technical skills is also an issue for human workers [2, 101]. Programming to the industrial robot and managing translation in the new jobs are difficult tasks requiring a high level of technical skills.
2. Adoption of advanced technology is required more time and effort from the side of the human workers. Customized software-connected factories, collaborative robotics, artificial intelligence, real-time information, and the internet of things must be adopted for industry 5.0 [50, 111–113].
3. Advanced technologies are required investments. UR Cobot is not coming cheap. Training the human workers for new jobs is bringing extra costs. The companies are found it difficult to upgrade the production lines for industry 5.0 [114]. Adopting Industry 5.0 is expensive as it requires smart machines and highly skilled employees to increase productivity and efficiency.
4. Security is a challenge for Industry 5.0 as it is critical to establish trust in ecosystems. The authentication is used in the industry is the scale to interact with various devices, to stand against the future quantum computing applications to deploy nodes of IoT [60, 94, 115]. Usage of artificial intelligence and automation in industry 5.0 are threats for the business, and therefore it is required to have trusted security for it [88, 116–118]. The applications of Industry 5.0 are focused on the ICT systems, and therefore it leads to strict security requirements to prevent the security challenges.

## Lessons learned

**Fig. 4:**
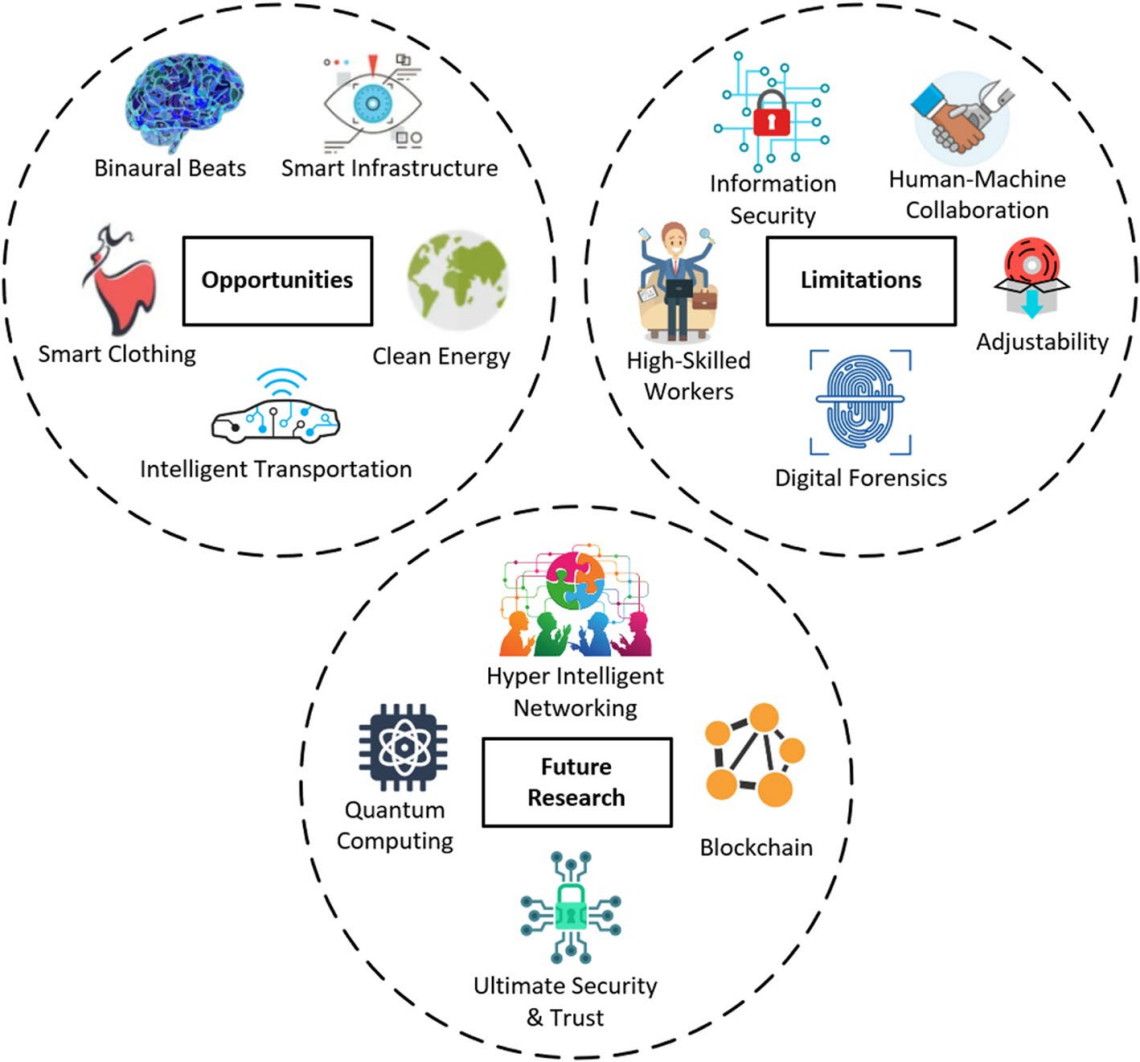
Opportunities, Limitations, and Future Research of Industry 5.0

The evolution of Industry 5.0 is done in the retail, healthcare, finance, manufacturing and other industries. The lessons learned are discussed from various studies, applications, and enabling technologies of Industry 5.0. Figure 4 illustrates the opportunities, limitations, and future research directions on industry 5.0.

### Definitions of industry 5.0

From definitions of Industry 5.0, it is being observed that it is an innovative production model where the focus is based on interaction among humans and machines [56, 119–122]. It is involved with leveraging collaboration among increased power and efficient machinery and innovative potential of human beings. The existing projects based on which industry 5.0 is conducted related to the optimization of artificial intelligence that leads to customized products [18, 46, 123–128]. There is the adoption of this industry at the international standards.

### Applications of industry 5.0

Industry 5.0 provides benefits for the industry for both the workers and society. There is also an increase in the competitiveness of the business and help attract the best talents [129–131]. Adoption of this industry supports technologies that make natural usage of the resources properly. Human robotics, such as Sophia, personifies dreams of the future of artificial intelligence [132, 133]. It helps in the decision making of humans and is supported by enabling technologies that help in revolutionize various sectors. Even various challenges are mentioned in this paper, like handling quantity of data, managing resources, and others.

### Enabling technologies

The enabling technologies of industry 5.0 are set for complex systems that can combine the technologies such as smart materials, human-machine interaction, big data analytics, cloud computing, and others [134]. Smart manufacturing and intelligence help reduce network traffic, facilitate transactions, and privacy, which helps the business use software resources to exchange data about the industrial sectors [135]. Blockchain technology is automated agreement processes among various stakeholders, while smart contracts manage security, authentication, and automated service-related actions [136, 137]. 6G network is expected to meet with the intelligent information standard that provides high energy efficiency, high reliability, plus capacity of traffic. Big data analytics is the enabling technology that helps manage a large amount of data [17, 116, 138]. Even the internet of things is an opportunity for industry 5.0 that can reduce operating costs by eliminating issues on the communication network, waste management, supply chain, production process optimization, and others.

### Limitations of industry 5.0

Acceptance of technology and trust in the technologies are critical. Adaptation of the technology to humans coincides with training people who are using the new technologies [139–143]. Current challenges are security, privacy, lack of skilled workers, time-consuming process, and large budget required. Adoption of industry 5.0 is required to follow industrial laws and regulations that can help to work together with smart machines plus cobots. Future directions for industry 5.0 are cognitive computing, human and machine interaction, and quantum computing.

## Future directions

Cognitive computing: This application aims to stimulate the thoughts of the human in processes into a computerized model [16, 144, 145]. Using the self-learning algorithms uses data mining, recognition of patterns, natural language, and others that the computer can read that the human brain will work.

Human and machine interaction refers to communication with interaction among humans and machines via the user interface. Natural user interfaces like gestures are used to gain attention as they allow humans to control the machines through intuitive and natural behaviours [86, 146–149]. It is the future direction for industry 5.0 as it helps to keep the humans at the centre of the system and technologies to build in. Even the user interface helps people understand people’s behaviour and motivations.

Quantum computing is a type of computation that can harness collective properties of the quantum states, like interference entanglement, to do the calculations. The devices are performed quantum computations which are defined as quantum computers [8, 150–153]. It is performing calculations focused on the probability of the object’s state before it is measured.

## Conclusion

From the study, it is concluded that the author started the work with definitions of industry 5.0 from the perspective of the industrial as well as academic communities. Even the applications have also been discussed that help better understand the features of industry 5.0, followed by a discussion of enabling technologies. Industry 5.0 concept is designed to make the efficiency of humans and machines correctly. Challenges are also presented in this paper that help manage the issues caused in industry 5.0. Future directions are discussed in this paper that should be handled better to use this industry shortly.

## Data Availability

The datasets used during the current study are available from the corresponding author on reasonable request.
